# Describing the characteristics and symptom profile of a group of urban patients experiencing socioeconomic inequity and receiving palliative care: a descriptive exploratory analysis

**DOI:** 10.1177/26323524241264880

**Published:** 2024-08-01

**Authors:** Harrison Moore, Cara Bablitz, Anna Santos Salas, Heather Morris, Aynharan Sinnarajah, Sharon M. Watanabe

**Affiliations:** Division of Palliative Care Medicine, Department of Oncology, Faculty of Medicine & Dentistry, College of Health Sciences, University of Alberta, 8440 112 Street NW, Edmonton, AB T6G 2B7, Canada; Department of Family Medicine, Faculty of Medicine & Dentistry, College of Health Sciences, University of Alberta; Family Medicine, Royal Alexandra Hospital, Edmonton, AB, Canada; Faculty of Nursing, College of Health Sciences, University of Alberta, Edmonton, AB, Canada; Faculty of Nursing, College of Health Sciences, University of Alberta, Edmonton, AB, Canada; Division of Palliative Medicine, Department of Medicine, Faculty of Health Sciences, Queen’s University, Kingston, ON, Canada; Department of Symptom Control and Palliative Care Cross Cancer Institute, Edmonton, AB, Canada; Division of Palliative Care Medicine, Department of Oncology, Faculty of Medicine & Dentistry, College of Health Sciences, University of Alberta, Edmonton, AB, Canada

**Keywords:** health status disparities, homelessness, Indigenous Peoples, palliative care, social determinants of health, socioeconomic factors

## Abstract

**Background:**

Individuals experiencing socioeconomic inequity have worse health outcomes and face barriers to palliative and end-of-life care. There is a need to develop palliative care programs tailored to this underserved population.

**Objectives:**

To understand the characteristics and symptom profiles of a group of urban patients experiencing socioeconomic inequity and receiving palliative care.

**Design:**

Descriptive exploratory analysis of a patient dataset. The patient dataset was generated through a pilot research study with patients experiencing socioeconomic inequity and life-limiting illness who received a community-based palliative care intervention.

**Methods:**

The intervention took place over 1 year in the Palliative Care Outreach and Advocacy Team, a community-based urban palliative care clinic in Edmonton, Alberta, Canada, serving persons experiencing socioeconomic inequity. Participants had to be at least 18 years of age, be able to communicate in English, require palliative care for a life-limiting illness, and be able to consent to inclusion in the study.

**Results:**

Twenty-five participants were enrolled. Participants predominantly identified as male and Indigenous, experienced poverty and housing instability, and had metastatic cancer. Our participants rated their pain, shortness of breath, and anxiety as more severe than the broader community-based palliative care population in the same city. Most patients died in inpatient hospices (73%).

**Conclusion:**

Our analysis provides an in-depth picture of an understudied, underserved population requiring palliative care. Given the higher symptom severity experienced by participants, our analysis highlights the importance of person-centered palliative care. We suggest that socioeconomic inequity should be considered in patients with life-limiting illnesses. Further research is needed to explore palliative care delivery to those facing socioeconomic inequity.

## Introduction

Recently, there has been an increase in research focusing on palliative care for individuals experiencing socioeconomic inequity. Socioeconomic inequity refers to disparities in access to resources, opportunities, and outcomes, including health outcomes, among different socioeconomic groups within a society. Socioeconomic status is influenced by income, education, employment, wealth, and place of residence.^1^ The World Health Organization writes that ‘health inequities are systematic differences in the health status of different population groups’.^2^ While still uncommon, there are an increasing number of palliative care programs operating outside of traditional healthcare settings that provide care to individuals experiencing socioeconomic inequity.^3^ This ranges from community-based care teams that provide care where a person resides, including in shelters for those experiencing houselessness, to inpatient hospices serving those without housing or living with substance use disorder.

Previous research with individuals experiencing socioeconomic inequity, specifically houselessness, and receiving palliative care involved qualitative interviews to better understand an individual’s experience of illness and views of the healthcare system or the experience and views of palliative care providers.^4–8^ Other retrospective studies, such as chart reviews, aimed to better understand the characteristics and challenges faced by individuals experiencing housing instability while requiring palliative care.^9,10^ Complexity of care needs, both physical and psychosocial, barriers to care including difficulty accessing care or finding an appropriate location for care, and challenges associated with ongoing substance use are frequently observed findings. These findings highlight the need for comprehensive, person-centered palliative care programs.

Furthermore, there is a growing body of research regarding the provision of palliative care to Indigenous Peoples.^11–13^ Key themes include the importance of culturally safe care and family involvement, increased local capacity building to deliver palliative care close to home, and institutional barriers to receiving palliative care. Past research, however, has not discussed symptom severity for those experiencing socioeconomic inequity and the resulting urgency for palliative care.

Individuals who experience socioeconomic inequity have worse health outcomes than the general population.^14^ Indigenous Peoples in Canada, as well as countries such as the United States and Australia, are more likely to experience socioeconomic inequity.^15–17^ Indigenous scholars explain that socioeconomic inequity among Indigenous Peoples is a result of a systemic denial of the means required to advance socioeconomic status.^18^ This socioeconomic inequity is evidenced ‘in high rates of unemployment, scarce economic opportunities, poor housing, low literacy, and educational attainment as well as meager community resources’.^18^ In Canada, colonization and assimilation practices separated Indigenous Peoples from their Indigenous languages, traditions, and healing practices. This reduced access to culture, combined with experiences of discrimination and marginalization, has led to a lack of trust in the healthcare system and has greatly impacted the health of Indigenous Peoples.^19^

Housing instability is independently associated with a higher incidence of cancer,^20^ as well as cardiovascular,^21,22^ lung,^23^ and liver^24^ diseases. Life expectancy for those who are experiencing houselessness or are precariously housed is significantly lower than for the population at large.^25–29^ Precarious housing refers to unmet core housing needs, meaning the home needs repairs, is unaffordable, or is unsuitable.^30^ Reasons for these poorer health outcomes are multifactorial; they include higher rates of substance use disorder,^31^ dangerous living conditions,^32–34^ and barriers to accessing healthcare resources.^6,35–39^ Considering these factors, it is not surprising that those experiencing houselessness and housing instability tend to use the emergency room and acute care services at a higher level than the general population.^40–43^

Due to historical and contemporary colonial landscapes, Indigenous Peoples experience higher rates of socioeconomic inequity and their health outcomes are worse than the general population.^13,44^ Indigenous Peoples suffer a disproportionate burden of mental illness,^45^ poorer cancer outcomes,^46^ as well as an overall shorter life expectancy when compared to non-Indigenous People.^44^ Indigenous Peoples are overrepresented in the precariously housed population in Canada, the United States of America, Australia, and New Zealand.^47–50^ Against this backdrop, the Government of Canada stated one of its four priority areas for palliative care to be ‘measures to facilitate equitable access to palliative care across Canada, with a closer look at underserviced populations’, which include unhoused and Indigenous individuals.^51^

In this paper, we describe the clinical profile of a sample of individuals with life-limiting disease experiencing socioeconomic inequity. Adopting an intersectional perspective,^52^ we recognize that the characteristics and experiences of each individual are unique and that multiple reasons exist for their experienced oppression. These individuals participated in a community-based pilot research study that sought to improve access to palliative care for the study population through a palliative care intervention.^53^ In order to identify potential disparities, we compared the clinical profile of the patient dataset generated through the pilot study with the population served by the broader palliative care program in the same city.

## Methods

### Design and setting

This is a descriptive exploratory secondary analysis of a patient dataset, written in accordance with the Strengthening the Reporting of Observational Studies in Epidemiology (STROBE) checklist, which is included in Supplemental Materials. Data were collected prospectively through a pilot research study that examined the impact of a community-based palliative care intervention on the experiences and multidimensional needs of patients experiencing socioeconomic inequity and living with life-limiting illnesses.^53^ The pilot research study followed a community-based participatory research methodology.^53^ The study took place in partnership with the Palliative Care Outreach and Advocacy Team (PCOAT), a community-based palliative care clinic in Edmonton, a city with a census metropolitan area population of nearly 1.5 million^54^ and the capital of the Canadian province of Alberta. PCOAT is situated in central Edmonton and operates within the Indigenous Wellness Clinic, close to community organizations. It provides supports to individuals, both Indigenous and non-Indigenous, experiencing socioeconomic inequity. This includes those who are precariously housed, living below the poverty line, or who have an active or recent history of substance use disorder.^55^ It provides culturally informed palliative care to patients in the community and collaborates with other palliative care services as required. In addition to seeing patients in the clinic, team members also visit patients wherever they reside.

### Study participants and recruitment

The intervention implemented in the pilot research study consisted of a part-time registered nurse who worked alongside a family physician specialized in palliative care (one of the study principal investigators and coauthor, CB). The nurse was involved in complex care coordination and follow-up care of consenting individuals. All participants received palliative care through PCOAT. Some participants were referred to the clinic by other healthcare providers, some by community support services, and some self-referred. Healthcare providers in the clinic, not involved in the study, identified patients eligible for participation in the study. Consenting participants had to be at least 18 years of age, be able to communicate in English, be able to consent to participation in a research study, and have a life-limiting illness. The intervention phase lasted from 23 January 2019 to 22 January 2020.

### Data collection

At the time of enrollment, demographic and clinical data were collected from each participant in addition to income and housing status. Data included participants’ primary diagnosis and comorbidities, and data from routinely used palliative care tools. These tools included the Edmonton Symptom Assessment Scale-revised (ESAS-r), Mini-Mental State Examination (MMSE), CAGE questionnaire, which is a screening tool for alcohol use disorder, and Palliative Performance Scale (PPS).^56–60^ Assessment with the aforementioned palliative care tools occurred during initial consultation with PCOAT. When assessment tools had been completed more than once, the most recent scores prior to or on the day of enrollment were used. One participant did not complete the MMSE, otherwise the dataset was complete. Using patient-reported comorbidities, an updated Charlson Comorbidity Index was calculated, excluding primary life-limiting diagnoses from the calculation as described by Quan *et al*.^61^ Over the course of the study period, through access to electronic medical records, emergency room visits and location of death were also recorded. Information concerning emergency room visits by each participant in the 12 months before study enrollment was also recorded. Participation in the study either ended when the study finished or when a patient died. All demographic and clinical data were extracted using REDCap©, a secure digital application to manage online data.

### Data analysis

To obtain a clinical profile of the study sample, we conducted a descriptive statistical analysis of clinical and demographic data collected in the intervention phase. When possible, we compared indicators routinely collected from the general population served by the Edmonton Zone Palliative Care Program. This program provides palliative care to patients residing in Edmonton and surrounding communities. It provides specialist palliative care consultation in all healthcare settings, including the community, acute care, and cancer center, in addition to a tertiary palliative care unit and inpatient hospices.^62^ Patients seen through the program undergo a comprehensive clinical assessment, including the tools named above. In our comparison, we focused on data collected at the time of initial consultation from patients seen at home by the Edmonton Zone Palliative Community Consult Team (EZPCCT) from 1 April 2019 to 31 March 2020. We elected to compare our study participants against patients seen by the community consult team as these patients also resided in the community at the time of consultation and had a mix of both cancer and non-cancer life-limiting diagnoses. Statistical analysis for comparison was not performed due to the small study sample size.

### Ethics approval

Ethics approval for the pilot research study was granted by the Health Research Ethics Board of Alberta Cancer Committee (Ethics ID: HREBA.CC-17-0255). Initial approval was received on 8 January 2018. Ethics approval for the intervention phase was received on 14 November 2018. Written consent was obtained from all study participants, with ongoing verbal consent obtained throughout the duration of the study.

## Results

### Participant characteristics

A total of 25 individuals were enrolled in the study (Table 1).^53^ The first and last participants were enrolled on 23 January 2019 and 20 November 2019, respectively. The median age of study participants was 59 years, and 40% (*n* = 10) were living with a non-cancer life-limiting illness. Types of cancer included colorectal, esophageal, fallopian tube, gastric, lung, melanoma, oropharyngeal, and pancreatic. Male patients made up 64% (*n* = 16) of our study population. In terms of ethnicity, 60% (*n* = 15) self-identified as Indigenous, with the remaining 40% (*n* = 10) being non-Indigenous. All patients reported that English was their primary language. Regarding housing, 48% (*n* = 12) were considered to be in stable housing at the time of study entry, 44% (*n* = 11) were precariously housed, and 8% (*n* = 2) were without housing. Regarding net financial income, 40% (*n* = 10) had an annual income of Canadian dollars (CAD) 0–20,000, while the remaining 60% (*n* = 15) had an income of CAD 20,000–40,000. However, most participants in this income category reported an income marginally higher than CAD 20,000. Sixty percent (*n* = 15) of study participants either had a history of, or were currently living with, a substance use disorder, while 52% (*n* = 13) had a CAGE score greater than or equal to two, suggesting a history of current or previous high-risk alcohol use (Table 2).

**Table 1.** Overview of study sample for study pilot palliative care interaction for people experiencing homelessness, Edmonton, Alberta, 2019–2020^53^

Descriptor	*n*
Sample size	25
Age, average (range), in years	57.3 (39–84)
Ethnicity	
Indigenous^a^	15
Caucasian	10
Sex; *n*	
Male	16
Female	9
Diagnosis	
Advanced cancer	15
Other advanced condition:	10
Liver failure (2)	
Advanced lung disease (6)	
Advanced kidney disease (1)	
Other (1)	
Income	
No income	1
CAD <20,000	9
CAD 20,000–CAD 40,000^b^	15
Housing status	
Stable housing	12
Precarious housing	11
No housing	2
Study intervention	
Length of stay in study, average (range), in months	5.5 (0.25–12)
Study RN interactions per participant, average (range)	9 (3–32)

a Refers to First Nations, Métis or Inuit people in Canada.

b Most had an annual income slightly above CAD 20,000.

CAD, Canadian dollars; RN, registered nurse.Source: Reprinted from Salas et al.^53^ Copyright (2023), with permission from The Public Health Agency of Canada (https://doi.org/10.24095/hpcdp.43.8.02).

**Table 2.** Additional participant characteristics.

Number of participants; *n*	25
Updated Charlson Comorbidity Index^a^; *n* (%)	
0	10 (40)
1	3 (12)
⩾2	12 (48)
Reported history of substance use disorder; *n* (%)	15 (60)
CAGE Questionnaire^b^ ⩾ 2; *n* (%)	13 (52)
Folstein Mini-Mental Status Exam^c,d^; average (range)	27.3 (22–30)

a Updated Charlson Comorbidity Index: a method of categorizing comorbidities of patients.

b CAGE: Screening tool for alcoholism (score ⩾ 2 suggests a history of current or previous high risk alcohol use).

c Folstein Mini-Mental State Exam: test of cognitive function, scores range from 0 to 30.

d Folstein Mini-Mental State Exam data were not available for one participant.

### Study outcomes

The average length of time in the study for all participants was 5.5 months (Table 3). Over the course of the study period, 11 participants died. No patients were lost to follow-up over the study period. There were no dropouts. Deceased participants were enrolled for an average of 2.1 months in the study. Eight of the eleven deceased participants (83%) died in inpatient hospices, while the remaining three deceased participants (27%) died in hospital. There was an observed decrease in emergency room utilization on a per-month basis. Participants’ emergency room utilization in the 12-month period before study enrollment was 0.48 visits per month, compared to their emergency room utilization during their time in the study at 0.31 visits per month. Among the 11 deceased participants, 6 visited the emergency room in the final 30 days of life; however, no participant visited more than once.

**Table 3.** Participant outcomes.

Descriptor	Deceased^a^ (*n* = 11)	Not deceased (*n* = 14)	All participants (*n* = 25)
Age (years); median (range)	59 (47–84)	56.5 (39–66)	59 (39–84)
Months enrolled in study; average (range)	2.1 (0.2–7.7)	8.2 (2.1–12.0)	5.5 (0.2–11.9)
ER visits per month before enrollment; average (range)^b^	0.51 (0–1.50)	0.43 (0–1.58)	0.48 (0–1.58)
ER visits per month after enrollment; average (range)	0.42 (0–1.43)	0.29 (0–1.81)	0.31 (0–1.81)
Place of death; *n* (%)			
Hospice	8 (73)	–	–
Hospital	3 (27)	–	–
Number of ER visits in the 30 days before death; *n* (%)			
0 visits	5 (45)	–	–
1 visit	6 (55)	–	–

a Participants deceased by the end of the study period.

b Based on the 12 month period before study enrollment.

ER, emergency room.

### Comparisons to broader palliative care population

Of the 1249 patients seen in the community by the EZPCCT between 1 April 2019 and 31 March 2020, 21.1% had a non-cancer diagnosis, which is lower than the 40% seen in the study participants (Table 4). While our study dataset of 25 participants is too small to allow for comparative statistical analysis, the study participants had higher symptom severity when considering multiple symptoms. Specifically, the average ESAS-r pain scores of the study participants were 2.6 points higher when compared to a broader group of patients residing in the community receiving palliative care. Additionally, average ESAS-r scores for shortness of breath and anxiety were 2.5 points and 2.4 points higher, respectively. This higher symptom severity was seen in a group of patients who collectively had a higher performance status. Our study group’s average PPS score was 62.0 compared to 42.9 for the broader group of community-based palliative care patients. This difference of 19.1 points suggests a higher level of function in our study participants.

**Table 4.** Comparison of PCOAT^a^ and EZPCCT^b^ patients.

Descriptor	PCOAT patients			EZPCCT patients (*n* = 1249)
	Deceased^c^ (*n* = 11)	Not deceased (*n* = 14)	All (*n* = 25)	
Non-cancer (%)	27.3	50.0	40.0	21.1
ESAS-r^d,e^ (average)				
Pain	5.3	5.4	5.3	2.7
Tiredness	5.9	6.4	6.2	6.8
Drowsiness	6.3	4.2	5.2	4.3
Nausea	1.3	1.8	1.5	0.9
Appetite	5.5	4.8	5.2	6.2
Shortness of breath	5.0	3.8	4.3	1.8
Depression	3.1	4.2	3.7	2.2
Anxiety	3.5	5.7	4.7	2.3
Wellbeing	6.8	5.5	6.1	4.9
PPS^f^(average)	57.2	65.7	62.0	42.9

a Palliative Care Outreach and Advocacy Team, study participants.

b Edmonton Zone Palliative Community Consult Team, community-based patients.

c Participants deceased by the end of the study period.

d ESAS-r: Edmonton Symptom Assessment System–revised (0 = absence of the symptom/best and 10 = worst possible severity).

e ESAS-r not available for one participant in the ‘Not Deceased’ subgroup.

f PPS: Palliative Performance Scale (0 = death, 100 = normal ambulation, normal activity and work, no evidence of disease, full self-care, normal intake, and full conscious level).

## Discussion

### Summary of findings

The participants in our study consisted of 25 individuals experiencing socioeconomic inequity, living with a life-limiting illness, and residing in an urban setting. The median age at enrollment of the participants who died, 59, was similar to that described in a retrospective study focusing on individuals experiencing houselessness and receiving palliative care in Toronto, Ontario, where the median age of death was 60.^10^ The median age at death of the study participants is well below the 2019 average life expectancy in Canada of 82.^63^ It is also well below the age at death of patients who received specialist palliative care in the neighboring city of Calgary, Alberta, where 64% of patients were 71 years or older when they died.^64^ It is noted that the hospital death rate in this study was only 27%, which is much lower than population hospital death rates (e.g. 42% of all deaths in Calgary occurred in acute care hospitals). This result aligns with previous research demonstrating reduced acute care hospital use for patients enrolled in a community-based palliative care program.^65^

The majority of study participants (60%) were Indigenous, in keeping with research suggesting that, due to the colonial effects on the determinants of health, Indigenous Peoples are overrepresented in those experiencing socioeconomic inequity in Canada.^15^ Of note, many study participants under the age of 65 qualified for provincial income assistance for severe medical disability; if this was arranged prior to enlisting in the study, it placed their income between CAD 20,000 and 25,000. For reference, in 2019 the average income in Alberta for an individual 15 years of age and older was CAD 62,600, while the median income was CAD 47,300.^66^ In addition to income disparity, over half of our participants were either without housing or precariously housed. Of those who were considered to be in stable housing, many had previously experienced housing instability or houselessness, and two had found stable housing with the assistance of PCOAT.

When examining the symptoms of study participants who experience socioeconomic inequity, symptom severity was higher compared to the general population receiving community-based palliative care consultation. On average, pain, shortness of breath, and anxiety were rated at 2.6, 2.5, and 2.4 points higher on ESAS-r, respectively, when compared to patients seen by the EZPCCT. What constitutes minimal clinically important differences in ESAS-r scores has been widely studied, and the differences seen in our study are greater than the proposed values that range from 0.8 to 2.3 depending on specific symptom and method of analysis used.^67,68^ These more severe symptoms are in a group whose average PPS score was 19.1 points higher than patients seen by the EZPCCT, 62.0 compared to 42.9. A score of 60 describes a person who is still ambulatory, albeit with limitations, whereas a score of 40 describes a person who spends most of their time in bed. While we do not have mortality data with which to compare an individual’s PPS score, the score has been shown to have significant prognostic value, with a higher score being associated with a longer prognosis.^60,69^ This suggests that participants in our study may be living with severe symptoms earlier in the trajectory of their disease. This higher symptom severity was seen in both the decedent and non-decedent participants.

### Implications for practice, research, and policymaking

Despite an increase in research into palliative care of individuals experiencing socioeconomic inequity, they remain a population that is understudied and one that experiences significantly poorer health outcomes related to multiple systemic inequities.^70,71^

Our research findings suggest patients requiring palliative care who are underserved as a result of either low socioeconomic status, housing instability, substance use disorder, systemic racial inequity, or a combination thereof are likely to experience comparable if not more severe symptoms through their disease trajectory. This finding, coupled with the well-documented challenges such individuals face accessing health care through conventional health system pathways,^72^ reinforces the need for further support and the creation of person-centered palliative care services. These services should provide care in a unique manner that responds to the needs and priorities of this population. As an example, PCOAT collaborates with a number of different agencies to assist in the provision of food, clothing, shelter, income assistance, cultural support, and family reunification as required by the individual receiving care.^55^

Of our group of patients, 60% identified as Indigenous. Our program was run out of an Indigenous Wellness Clinic and led by an Indigenous physician (CB), which may have resulted in higher rates of referral for Indigenous patients. However, the overrepresentation of Indigenous Peoples in this study aligns with the socioeconomic outcomes of Indigenous Peoples in Canada. There is a disproportionate rate of chronic disease burden and higher mortality rates in Indigenous Peoples when compared to non-Indigenous People.^44–46^ It is well documented that the historical implications of colonization, residential schools, multigenerational trauma, and systemic anti-Indigenous racism have greatly impacted the health and well-being of Métis, Inuit, and First Nations Peoples in Canada.^19^ Indigenous identity further shapes the type of person-centered care needed in Canada for those experiencing socioeconomic inequity. Further exploration is needed to understand the additional challenges Indigenous Peoples face in accessing culturally informed and safe palliative care. Research priorities should be community-led and include collaboration, consultation, and engagement with all Indigenous Peoples, including, as in our study, urban Indigenous groups. Efforts are needed to improve access to palliative and end-of-life care and services with a wholistic health approach for all Indigenous Peoples.

### Strengths and limitations

Our descriptive exploratory analysis is based on data prospectively collected through a pilot research study involving a population experiencing socioeconomic inequity and living with life-limiting illness. We were able to draw novel insight into the severity of their symptoms. These findings support the calls for radical health system changes to address the needs of those experiencing socioeconomic inequity.

The primary study followed a community-based participatory research approach. Thus, the study sample was too small to perform advanced statistical analysis. However, the differences identified point to clinical disparities in the profile of our study sample when compared with the larger palliative care population in the same region. The study data point to the potential impact of a dedicated palliative care program that alleviates both severe socioeconomic inequities and complex health needs when serving this highly underserved population. As all our patients lived in the same city, further research would be helpful to explore patient characteristics and symptom burden in other locations. As well, ability to provide consent was a requirement for study inclusion that may have led to a study group with higher performance status due to the exclusion of those too unwell to consent to study participation. It is also not known whether patients with severe socioeconomic inequities who did not enroll in palliative care programs had comparable or different symptom scores. It might be that palliative care referral is only initiated when this population has much higher symptom scores than the general population. With that said, we believe that the information gathered in the study adds to our understanding of the experience of people living with a life-limiting illness in systemically oppressed circumstances by showing increased symptom severity.

## Conclusion

We followed a group of urban patients experiencing socioeconomic inequity and receiving palliative care services over the course of a year. Our descriptive analysis provides further insight into an understudied population facing multiple systemic inequities in Canada. We discovered that the severity of a number of symptoms associated with a life-limiting illness tended to be higher in our study population than in the broader palliative care population in our region. We hope that our study provides insight into the experience of this population and serves as a stepping stone for further research into the area.

## Supplemental Material

sj-docx-1-pcr-10.1177_26323524241264880 – Supplemental material for Describing the characteristics and symptom profile of a group of urban patients experiencing socioeconomic inequity and receiving palliative care: a descriptive exploratory analysis Supplemental material, sj-docx-1-pcr-10.1177_26323524241264880 for Describing the characteristics and symptom profile of a group of urban patients experiencing socioeconomic inequity and receiving palliative care: a descriptive exploratory analysis by Harrison Moore, Cara Bablitz, Anna Santos Salas, Heather Morris, Aynharan Sinnarajah and Sharon M. Watanabe in Palliative Care and Social Practice

## Declarations

**Ethics approval and consent to participate:** Ethics approval for the pilot research study was granted on 8 January 2018: The research study RES0035812 Symptom Relief and Palliative Care for Inner City Populations in Edmonton: A Pilot Intervention was approved by the Health Research Ethics Board of Alberta Cancer Committee (Ethics ID: HREBA.CC-17-0255). All participants provided written consent with ongoing verbal consent confirmed throughout the data collection process.

**Consent for publication:** Written informed consent for publication of their clinical details was obtained from all participants at the time of enrolment in the study.

**Availability of data and materials:** All data pertaining to the research reported in this article are securely stored in a research repository in the Faculty of Nursing at the University of Alberta. Due to ethical requirements, these data cannot be released. For data access requests, please contact Dr. Anna Santos Salas at avs@ualberta.ca. For data access requests pertaining to data from the Edmonton Zone Palliative Care Program, please contact: EZPCP@albertahealthservices.ca.
